# A Sex-Specific Role of Endothelial Sirtuin 3 on Blood Pressure and Diastolic Dysfunction in Female Mice

**DOI:** 10.3390/ijms21249744

**Published:** 2020-12-21

**Authors:** Heng Zeng, Xiaochen He, Jian-Xiong Chen

**Affiliations:** Department of Pharmacology and Toxicology, School of Medicine, University of Mississippi Medical Center, Jackson, MS 39216, USA; xhe2@umc.edu (X.H.); jchen3@umc.edu (J.-X.C.)

**Keywords:** Sirtuin 3, endothelial, female, diastolic dysfunction, high-fat-diet-induced obesity

## Abstract

Background: Heart failure with preserved ejection fraction (HFpEF) is characterized by a diastolic dysfunction and is highly prevalent in aged women. Our study showed that ablation of endothelial Sirtuin 3 (SIRT3) led to diastolic dysfunction in male mice. However, the sex-specific role of endothelial SIRT3 deficiency on blood pressure and diastolic function in female mice remains to be investigated. Methods and Results: In this study, we demonstrate that the ablation of endothelial SIRT3 in females elevated blood pressure as compared with control female mice. Diastolic function measurement also showed that the isovolumic relaxation time (IVRT) and myocardial performance index (MPI) were significantly increased, whereas the E’ velocity/A’ velocity (E’/A’) ratio was reduced in the endothelial-specific SIRT3 knockout (SIRT3 ECKO) female mice. To further investigate the regulatory role of endothelial SIRT3 on blood pressure and diastolic dysfunction in metabolic stress, SIRT3 ECKO female mice were fed a normal diet and high-fat diet (HFD) for 20 weeks. The knockout of endothelial SIRT3 resulted in an increased blood pressure in female mice fed with an HFD. Intriguingly, SIRT3 ECKO female mice + HFD exhibited impaired coronary flow reserve (CFR) and more severe diastolic dysfunction as evidenced by an elevated IVRT as compared with control female mice + HFD. In addition, female SIRT3 ECKO mice had higher blood pressure and diastolic dysfunction as compared to male SIRT3 ECKO mice. Moreover, female SIRT3 ECKO mice + HFD had an impaired CFR and diastolic dysfunction as compared to male SIRT3 ECKO mice + HFD. Conclusions: These results implicate a sex-specific role of endothelial SIRT3 in regulating blood pressure and diastolic function in mice. Deficiency of endothelial SIRT3 may be responsible for a diastolic dysfunction in aged female.

## 1. Introduction

Heart failure (HF) is a highly prevalent disease, driven by aging, hypertension, diabetes mellitus, obesity, and ischemic heart disease [1]. Among HF patients, approximately one half of the patients are diagnosed with heart failure with preserved ejection fraction (HFpEF) with a characteristic of diastolic dysfunction [2,3,4,5]. HFpEF is strongly associated with advanced age and metabolic comorbidities, as well as microvascular dysfunction [6,7,8]. Yet, the pathophysiological mechanism of the development of diastolic dysfunction and HFpEF and effective therapies remain to be explored.

Over the past decade, compelling epidemiological data and clinical studies indicate a distinct gender distribution where women constitute about two thirds of the HFpEF patients [9,10,11]. The sex difference in the prevalence and clinical outcomes is mostly attributed to the concentric left ventricular remodeling during hypertension, increased ventricular and arterial stiffness, and the lack of the protective effect of estrogen after menopause [8,11]. However, other genetic factors and post-translational modification involved in the sexual dimorphism have not been fully understood. A recent study demonstrates that the expression of Sirtuin 1 and Sirtuin 3 (SIRT3) was significantly decreased in the old female hearts when compared to young women, associated with a significant increase in the infiltration of cardiac macrophages and pro-inflammatory cytokines, whereas in male human hearts, these changes were not observed [12]. This study suggests that SIRT3 may play a role in the sex differences related to aging.

Sirtuins are a family of NAD+-dependent type III histone deacetylases [13]. Among the Sirtuin family, SIRT3 is primarily localized in the mitochondria [13]. It has been associated with long lifespans in humans and rodents [14,15,16]. The level of SIRT3 was decreased in the heart of db/db diabetic mice in association with microvascular rarefaction [17], as well as in diabetic and obese patients [18,19]. Diabetes and obesity are commonly associated with HFpEF, where up to 50% of patients with HFpEF are affected by obesity or diabetes [20]. Particularly for obesity, women have considerably more adipose tissue than men with obesity, which may contribute to the greater effect of obesity on women than men [20,21]. Our previous study demonstrates that ablation of endothelial SIRT3 led to the altered endothelial cell (EC) metabolism and diastolic dysfunction in male mice [6], suggesting a cell-specific effect of SIRT3 on EC glycolytic metabolism, and coronary microvascular and diastolic dysfunction. However, the sex-specific role of endothelial SIRT3 deficiency on blood pressure and diastolic function in female, especially obese mice, has not been investigated.

In the present study, we utilized the unique endothelial-specific SIRT3 knockout (SIRT3 ECKO) male and female mice to investigate the sex-specific role of endothelial SIRT3 on blood pressure and diastolic function in females. We found that the female SIRT3 ECKO mice had impaired diastolic function, along with increased blood pressure, as compared with their female control mice. The female SIRT3 ECKO mice fed a high-fat diet (HFD) exhibited impaired coronary flow reserve (CFR) and more serve diastolic dysfunction. In addition, female SIRT3 ECKO mice fed an HFD showed impaired diastolic function as compared to their male littermates. These results implicate a sex-specific role of endothelial SIRT3 in regulating blood pressure and diastolic function in mice.

## 2. Results

### 2.1. Specific Knockout of Endothelial SIRT3 Elevates Blood Pressure in the Female Mice

**Figure 1:**
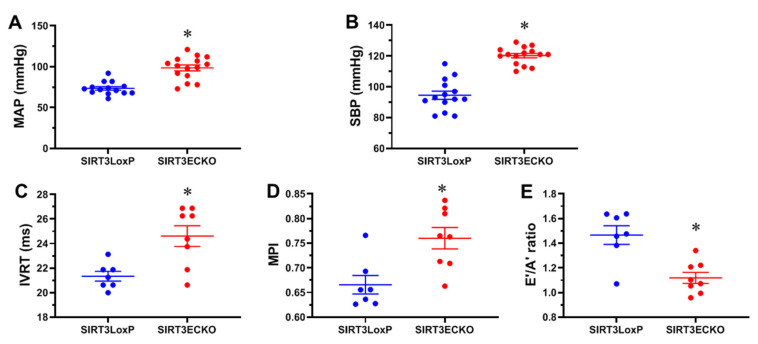
Loss of endothelial SIRT3 elevates blood pressure and impairs diastolic function in the female mice. (**A**,**B**) Mean arterial blood pressure (MAP) and systolic blood pressure (SBP) are elevated in the female endothelial-specific SIRT3 knockout (SIRT3 ECKO) mice when compared to SIRT3 LoxP mice. N = 14–15 mice. (**C**,**D**) Isovolumic relaxation time (IVRT) and myocardial performance index (MPI) are increased in the female SIRT3 ECKO mice when compared to SIRT3LoxP mice. (**E**) The E’/A’ ratio is decreased in the female SIRT3 ECKO mice when compared to SIRT3LoxP mice. N = 7–8 mice. * *p* < 0.05 vs. SIRT3 LoxP.

To first characterize the phenotype of the female SIRT3 ECKO mice, the blood pressure was measured by the tail-cuff method and compared between female SIRT3 LoxP and ECKO mice. Female SIRT3 ECKO mice exhibited a significant increase in the mean arterial pressure (MAP) and systolic blood pressure (SBP) when compared to the control mice (Figure 1A,B).

### 2.2. Specific Knockout of Endothelial SIRT3 Impairs Diastolic Function in the Female Mice

Our previous study demonstrates that ablation of endothelial SIRT3 led to the altered endothelial cell (EC) metabolism and diastolic dysfunction in male mice [6], suggesting a cell-specific effect of SIRT3 on EC glycolytic metabolism, and coronary microvascular and diastolic dysfunction. We then assessed the diastolic function in the female mice. Interestingly, the female SIRT3 ECKO mice exhibited prolonged IVRT when compared to the SIRT3 LoxP female mice (Figure 1C). This resulted in an increase in MPI in the female SIRT3 ECKO mice (Figure 1D). In addition, the E’/A’ as measured by pulsed-wave tissue Doppler was significantly decreased (Figure 1E) in the SIRT3 ECKO mice. These data suggest that SIRT3 deficiency in endothelial cells impairs diastolic function in female mice.

### 2.3. Sex-Specific Effect of Endothelia SIRT 3 Deficiency on BP and Diastolic Function

**Figure 2:**
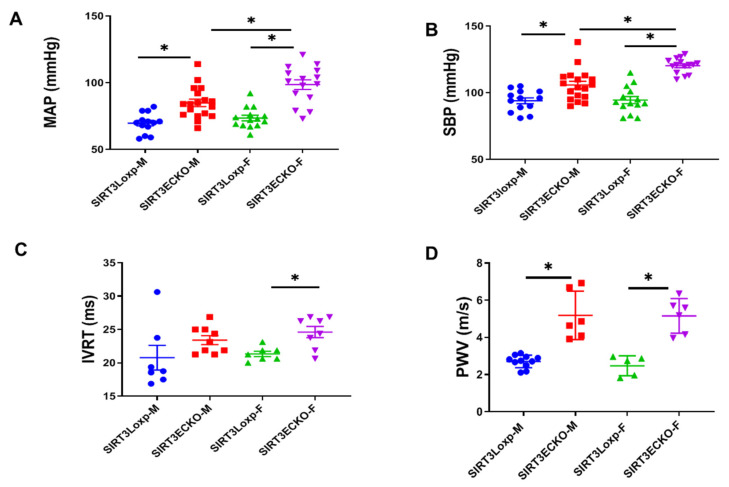
The role of endothelial SIRT3 deficiency on sex differences. (**A**,**B**) MAP and SBP are elevated in both male and female SIRT3 ECKO mice when compared to the corresponding SIRT3LoxP mice. However, female SIRT3 ECKO mice have higher blood pressure than the male mice. N = 13–18 mice. (**C**) IVRT is increased in the female SIRT3 ECKO mice when compared to the female SIRT3LoxP and male SIRT3 ECKO mice. N = 7–9 mice. (**D**) The arterial stiffness is significantly increased in both male and female SIRT3 ECKO mice when compared to the corresponding SIRT3LoxP mice. Female SIRT3 ECKO mice have similar pulse wave velocity (PWV) as the male SIRT3 ECKO mice. N = 5–11 mice. * *p* < 0.05.

To investigate whether endothelial SIRT3 plays a role on sex difference, the female SIRT3 LoxP and SIRT3 ECKO mice were compared to their male littermates. Similarly, SIRT3 deficiency led to increased MAP and SBP in male SIRT3 ECKO mice (Figure 2A,B). However, the blood pressure was significantly higher in the female SIRT3 ECKO mice than the male littermates (Figure 2A,B). In addition, the female SIRT3 ECKO mice had significantly longer IVRT than the female control mice, whereas the IVRT was not different between the male SIRT3 LoxP and SIRT3 ECKO mice (Figure 2C). Loss of endothelial SIRT3 increased arterial stiffness in both male and female mice, but there was no difference between the male and female SIRT3 ECKO mice (Figure 2D). These data suggest that SIRT3 deficiency has more profound effects on the blood pressure and diastolic function in the females than in the male mice.

### 2.4. Specific Knockout of Endothelial SIRT3 Exacerbates High-Fat-Diet-Induced Changes in Hemodynamics and Diastolic Function

**Figure 3:**
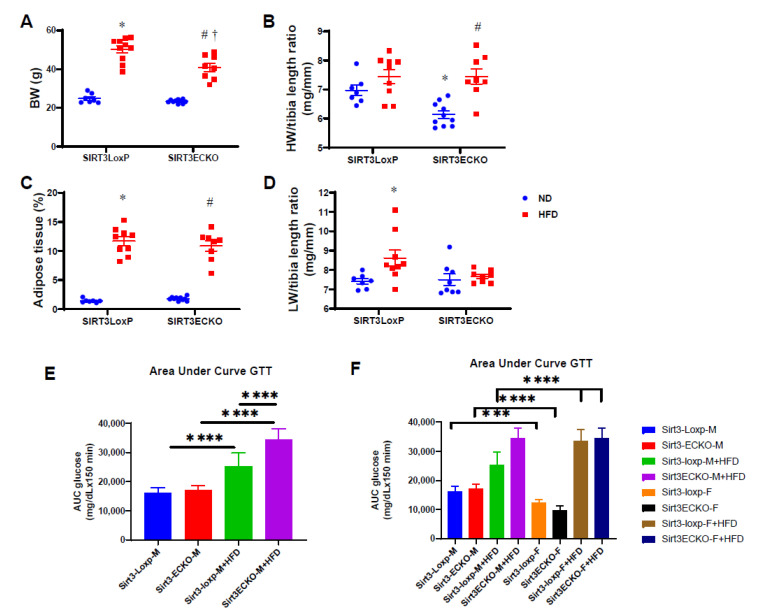
High-fat-diet (HFD)-induced obesity, cardiac, and lung growth. (**A**,**C**) HFD induced a significant increase in the body weight and percentage of adipose tissue in both SIRT3 LoxP and SIRT3 ECKO mice. (**B**) HFD induced cardiac hypertrophy only in female SIRT3 ECKO mice as evidenced by the increased heart weight (HW)/tibia length ratio. (**D**) By contrast, HFD caused increased lung growth only in female SIRT3 LoxP mice. N = 7–10 mice. * *p* < 0.05 vs. SIRT3 LoxP+normal diet (ND); # *p* < 0.05 vs. SIRT3 ECKO+ND; † *p* < 0.05 vs. SIRT3 LoxP+HFD. (E) Quantification of area under curve (AUC) for glucose tolerance test (GTT) in male SIRT3-Loxp mice and SIRT3 ECKO mice fed a ND or HFD (N = 8–10 mice). **** *p* < 0.0001. (F) Quantification of area under curve for glucose tolerance test in male and female SIRT3-Loxp mice and SIRT3 ECKO mice fed a ND or HFD (N = 7–9 mice). *** *p* < 0.001, **** *p* < 0.0001.

Obesity is commonly associated with endothelial dysfunction and HFpEF. To further investigate the role of SIRT3 on HFD-induced cardiovascular dysfunction, we fed the female mice a high-fat diet (HFD) for 20 weeks. As expected, the HFD induced a significant increase in the body weight and percentage of adipose tissue in both female SIRT3 LoxP and female SIRT3 ECKO mice (Figure 3A,C). HFD-fed mice resulted in a significant decline in E’/A’ ratio. Surprisingly, the HFD induced cardiac hypertrophy only in female SIRT3 ECKO mice as evidenced by an increased HW/tibia length ratio (Figure 3B). By contrast, the HFD caused increased lung growth only in female SIRT3 LoxP mice (Figure 3D). Quantification of the area under curve (AUC) for the glucose tolerance test (GTT) showed that male SIRT3 LoxP mice fed the HFD exhibited an impairment in the GTT as evidenced by an elevated AUC (Figure 3E). Male SIRT3 ECKO mice fed the HFD resulted in a further impaired GTT compared to that of SIRT3 LoxP mice + HFD (Figure 3E). Most intriguingly, female SIRT3 LoxP mice and SIRT3 ECKO mice had a better GTT as compared to that of male SIRT3 LoxP mice and SIRT3 ECKO mice under ND conditions (Figure 3F). By contrast, female SIRT3 ECKO mice fed the HFD exhibited a worse GTT as compared to that of male SIRT3 LoxP mice + HFD (Figure 3F), suggesting female SIRT3 ECKO mice had a more profound impaired GTT under an HFD.

**Figure 4:**
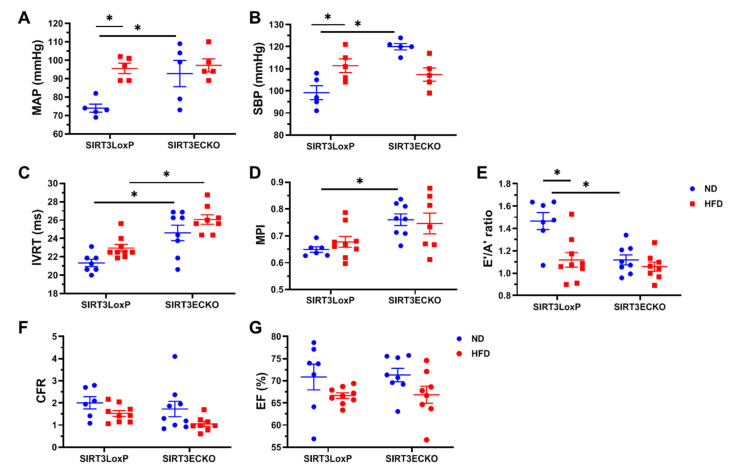
Loss of endothelial SIRT3 exacerbates HFD-induced changes in hemodynamics and diastolic function. (**A**,**B**) HFD results in a significant increase in the MAP and SBP in the female SIRT3 LoxP mice when compared to the ND-fed control mice. HFD does not further increase the blood pressure in the female SIRT3 ECKO mice. N = 5 mice. * *p* < 0.05 (**C**) HFD further prolongs the IVRT when compared to the SIRT3 LoxP mice fed a ND. * *p* <0.05 (**D**) HFD does not alter the MPI in either SIRT3 LoxP or SIRT3 ECKO mice. * *p* <0.05 (**E**) HFD leads to a significant decrease in the E’/A’ ratio in the female SIRT3 LoxP mice when compared to the ND-fed control mice, but HFD does not decrease the E’/A’ ratio in the female SIRT3 ECKO mice. N = 7–9 mice. * *p* < 0.05. (**F**) HFD does not significantly decrease the coronary flow reserve (CFR). N = 6–9 mice. (**G**) The ejection fraction (**E**,**F**) was not different between the female SIRT3 LoxP and SIRT3 ECKO mice when fed either ND or HFD. N = 7–9 mice.

An HFD resulted in a significant increase in the MAP and SBP in the female SIRT3 LoxP mice when compared to the normal diet (ND)-fed control mice (Figure 4A,B). By contrast, the HFD did not further increase the blood pressure in the female SIRT3 ECKO mice whose blood pressure had already been upregulated when fed a ND (Figure 4A,B). Similarly, the HFD resulted in a significant decrease in the E’/A’ ratio in the female SIRT3 LoxP mice when compared to the normal diet (ND)-fed control mice (Figure 4E), whereas the HFD did not further decrease the E’/A’ ratio in the female SIRT3 ECKO mice whose E’/A’ ratio had already been reduced when fed a ND (Figure 4E). Surprisingly, the HFD did not alter the MPI in either SIRT3 LoxP or SIRT3 ECKO mice (Figure 4D). However, the HFD exacerbated diastolic dysfunction in the female SIRT3 ECKO mice by further prolonging the IVRT when compared to the SIRT3 LoxP mice fed a ND (Figure 4C). The HFD tended to decrease the coronary flow reserve (CFR), but this was not statistically significant (Figure 4F). Interestingly, the ejection fraction (EF) was not different between the female SIRT3 LoxP and SIRT3 ECKO mice under either ND or HFD conditions (Figure 4G), suggesting a pre-existing HFpEF phenotype, which was exacerbated by the HFD in the female SIRT3 ECKO mice.

### 2.5. The Role of Endothelial SIRT3 Deficiency on Sex Differences in HFD-Induced Cardiovascular Dysfunction

**Figure 5:**
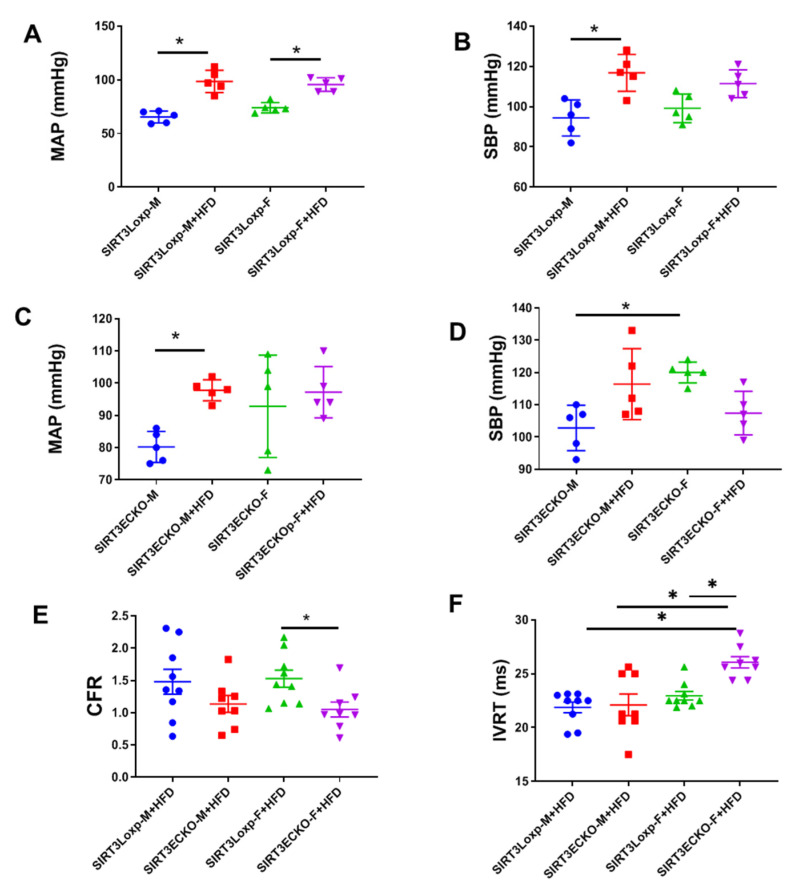
The role of endothelial SIRT3 deficiency on sex differences in HFD-induced cardiovascular dysfunction. (**A**,**B**) HFD increases the blood pressure in both male and female control SIRT3 LoxP mice. (**C**,**D**) HFD does not further increase blood pressure in female SIRT3 ECKO mice as observed in the male SIRT3 ECKO mice. N = 5 mice. * *p* < 0.05 (**E**,**F**) Female SIRT3 ECKO mice fed an HFD exhibit impaired CFR and diastolic dysfunction when compared to the female SIRT3LoxP mice. The HFD decreases CFR and increases IVRT in the female SIRT3 ECKO mice but not in the male SIRT3 ECKO mice fed an HFD. N = 8–9 mice. * *p* < 0.05

To further investigate the role of SIRT3 on sex differences in HFD-induced cardiovascular dysfunction, we compared the blood pressure and diastolic function between the male and female mice fed an HFD for 20 weeks. The HFD increased the blood pressure in both male and female control LoxP mice (Figure 5A,B), whereas in female SIRT3 ECKO mice, the HFD did not further increase blood pressure as observed in the male SIRT3 ECKO mice (Figure 5C,D). Female SIRT3 ECKO mice fed an HFD exhibited impaired CFR and diastolic dysfunction when compared to the female LoxP mice (Figure 5E,F). The HFD induced a decrease in CFR and an increase in the IVRT in the female SIRT3 ECKO mice, but not in the male SIRT3 ECKO mice (Figure 5E,F).

## 3. Discussion

The present study demonstrates that the female SIRT3 ECKO mice had impaired diastolic function, along with increased blood pressure, as compared with their female control mice. The female SIRT3 ECKO mice fed an HFD exhibited worse coronary flow reserve (CFR) and more severe diastolic dysfunction. In addition, female SIRT3 ECKO mice fed an HFD showed worse diastolic function as compared to their male littermates. These results implicate a sex-specific role of endothelial SIRT3 in regulating blood pressure and diastolic function in mice. Deficiency of endothelial SIRT3 may be responsible for the development of HFpEF in aging females.

The role of SIRT3 in hypertension has been reported. Dikalova et al. reported that the down-regulation of SIRT3 contributes to the pathogenesis of hypertension via inactivating SOD2 [22]. SIRT3 has also been shown to blunt hypertension-induced renal injury via suppression of the endothelial-to-mesenchymal transition [23]. These data suggest a cell type or genetic background-specific effect of SIRT3 [24]. Indeed, our previous study demonstrates that ablation of endothelial SIRT3 led to the altered endothelial cell (EC) metabolism and diastolic dysfunction in 12 month-old male mice [6,25], further suggesting a cell-specific effect of SIRT3 on EC glycolytic metabolism that plays a critical role in coronary microvascular and diastolic dysfunction that contributes to the development of HFpEF [26]. Endothelial dysfunction has contributed to the development of hypertension both in preclinical and clinic studies. However, the role of endothelial SIRT3 on hypertension in females has not been studied. As expected, loss of SIRT3 in endothelial cells resulted in an elevation of MAP and SBP in the female mice as compared to its female control mice, suggesting that endothelial SIRT3 does contribute to the regulation of blood pressure.

Hypertension is one of the major risk factors in HFpEF development and progression [27,28,29]. Therefore, we further assessed the diastolic function in the female SIRT3 ECKO mice. Not surprisingly, we found that the female SIRT3 ECKO mice exhibited impaired diastolic function as evidenced by prolonged IVRT, increased MPI, and decreased E’/A’ ratio. These data suggest that SIRT3 deficiency in endothelial cells also impairs diastolic function in female mice. To further investigate whether SIRT3 plays a role on sex difference, we compared the blood pressure and diastolic function in the female SIRT3 LoxP and SIRT3 ECKO mice to their male littermates. We found that although the loss of SIRT3 significantly increased the blood pressure and arterial stiffness in both male and female SIRT3 ECKO mice as compared to the SIRT3 LoxP mice, the blood pressure was significantly higher in female SIRT3 ECKO than the male SIRT3 ECKO mice. In addition, the female SIRT3 ECKO mice had significantly longer IVRT than the female control mice, whereas the IVRT was not different between the male SIRT3 LoxP and SIRT3 ECKO mice. These data suggest that SIRT3 deficiency has more profound effects on the blood pressure and diastolic function in the females than in the male mice. However, the underlying mechanism has not been fully understood.

Our present study showed that the HFD induced a decline in E’/A’ ratio, which indicates a diastolic dysfunction; however, no other diastolic parameters have been altered after being fed an HFD for 20 weeks. These results indicate that being fed an HFD alone may not be enough to produce a HFpEF phenotype, and a second hit such as deficiency of SIRT3 or hypertension is necessary. We then further investigated the effects of HFD-induced obesity on female SIRT3 ECKO mice and the potential sex differences. HFDs have significant effects on the blood pressure and diastolic function only in the female SIRT3 LoxP mice, as blood pressure has already been upregulated in the female SIRT3 ECKO when fed a ND. However, these effects were associated with significant cardiac hypertrophy only in the female SIRT3 ECKO mice. When compared to the male mice, similar effects were observed in the male SIRT3 ECKO mice. However, the female SIRT3 ECKO mice fed an HFD exhibited impaired CFR and diastolic dysfunction, which was not observed in the male SIRT3 ECKO mice fed an HFD. These data indicate that female SIRT3 ECKO mice may be a good rodent model for studying sex differences on hypertension, cardiac hypertrophy, and diastolic dysfunction in females. Interestingly, our present study showed that female SIRT3KO mice have small hearts as compared to the control female mice (Figure 3B). Similarly, HFD promoted lung development and growth in the control female mice, while female SIRT3 KO mice had little effects (Figure 3D). These results suggest that knockout of endothelial SIRT3 may retard organ growth under basic conditions and stress conditions in a cell/tissue type and sex-specific effect of SIRT3. The underlying mechanisms of these alterations remain unknown and warrant further investigation.

HFpEF patients often have endothelial dysfunction, dysregulation of the vascular tone, impaired angiogenesis, capillary rarefaction, and perivascular fibrosis formation [8,30]. Interestingly, these pathological processes are estrogen-mediated [8]. Although these changes were not assessed in the present study, loss of SIRT3 in female endothelial cells further elevated the blood pressure as compared to male mice, suggesting that SIRT3 may play a role in the estrogen-mediated signaling pathway. Our previous study demonstrated that knockout of endothelial SIRT3 impaired the angiogenic properties of ECs, including decreased EC tube formation, migration, and aortic sprouting. This was associated with an impaired hypoxic signaling pathway, as well as reduced CFR [6]. In our most recent study, we also revealed that knockout of endothelial SIRT3 disrupted endothelial glucose transport to cardiomyocyte, suggesting that endothelial SIRT3 may modulate heart performance by regulating microvascular function and delivery of the nutrients to the heart and controlling glucose uptake and transport to cardiomyocytes [31]. Consistent with these findings, our present study also showed that knockout of endothelial SIRT3 led to an impairment of the glucose tolerance test in HFD mice. Most interestingly, glucose tolerance is much better in female SIRT3 ECKO mice than that of male SIRT3 ECKO mice under normal diet conditions, while it became profoundly worse under HFD conditions. Taken together, these alterations in glucose metabolism may play a role somewhat in SIRT3-mediated sex differences in regulating blood pressure and diastolic function. Young women are better protected against oxidative stress due to their upregulated antioxidant defense mechanisms relative to age-matched men [32]. Recently, Lejri et al. reviewed the estrogen-SIRT3-dependent pathway in the brain and neurodegeneration [33]. Estrogen has been shown to increase electron transport chain activity and decrease ROS production, which is similar to the effect of SIRT3 on mitochondrial function [33]. These studies suggest that SIRT3 and estrogen may share a common pathway to regulate mitochondrial function and the antioxidant defense mechanism. However, whether the same mechanism plays a role in the endothelial cells and heart has not been well studied. Interestingly, a recent study demonstrates that old female hearts have a lower expression of SIRT3 and anti-oxidative enzyme SOD2 than in young female hearts [12]. This was associated with a significant increase in the infiltrating macrophages and pro-inflammatory cytokines in the old female hearts, which was not seen in the age-matched men [12]. This study further supports that SIRT3 is involved in a female sex-specific role in the aging hearts, although the underlying mechanisms by which SIRT3 contributes to these sex differences in the regulation of blood pressure and diastolic dysfunction remain unknown. Further studies are warranted to elucidate exactly the molecular mechanisms by which SIRT3 deficiency in EC causes the sex differences in cardiac diastolic dysfunction.

In conclusion, the present study demonstrates an important role of SIRT3 on sex differences in the endothelial cells in regulating blood pressure and diastolic function in mice. Deficiency of endothelial SIRT3 may be responsible for the development of diastolic dysfunction in aging females. Our study also provides a novel rodent model of studying sex differences on hypertension, cardiac hypertrophy, and diastolic dysfunction in females.

## 4. Methods

### 4.1. Animals and Experimental Design

All protocols were approved by the Institutional Animal Care and Use Committee (IACUC) of the University of Mississippi Medical Center (Protocol ID: 1280B) and were in compliance with the National Institutes of Health Guide for the Care and Use of Laboratory Animals (NIH Pub. No. 85–23, Revised 1996). 

The endothelial-specific SIRT3 knockout mice (SIRT3 ECKO) were generated by using the Cre-LoxP system as described in the previous studies [6,7]. Briefly, SIRT3^flox/flox^ mice (floxed exons 2 and 3) were crossbred with (B6.FVB-Tg(Cdh5-cre)7Mlia/J transgenic mice (Jackson Laboratory, Bar Harbor, ME, USA) expressing Cre recombinase specifically in vascular endothelial cells. The offspring Cdh5-Cre/SIRT3^flox/-^ heterozygous mice then mated with SIRT3^flox/flox^ to obtain the homozygous SIRT3 ECKO mice. Genotype was confirmed by tail DNA PCR analysis by using the following primers. Floxed SIRT3 allele primers: Forward 5′-TAC TGA ATA TCA GTG GGA ACG-3′, Reverse 5′-TGC AAC AAG GCT TTA TCT TCC-3′; WT SIRT3 primer: Forward 5′-CTT CTG CGG CTC TAT ACA CAG-3′; Cdh5-Cre transgene primers: Forward 5′-GCG GTC TGG CAG TAA AAA CTA TC-3′, Reverse 5′-GTG AAA CAG CAT TGC TGT CAC TT-3′; internal positive control Forward 5′-CTA GGC CAC AGA ATT GAA AGA TCT-3′ and internal positive control Reverse 5′-GTA GGT GGA AAT TCT AGC ATC ATC C-3′.

### 4.2. High-Fat-Diet-Induced Obesity Model

To induce obesity and cardiac dysfunction [34,35], 10 to 11 week-old male and female SIRT3 LoxP and SIRT3 ECKO mice were fed with either normal diet (ND) or high-fat diet (HFD, D12492, 60% kcal diet, Research Diets, New Brunswick, NJ, USA) for 20 weeks. The blood pressure, body weight (BW), heart weight (HW), adipose tissue weight, and lung weight (LW) were measured at the end of the experiment.

### 4.3. Glucose Tolerance Test (GTT)

After 20 weeks of HFD, the experimental mice were subjected to a glucose tolerance test (GTT) using the procedure described previously [34,35]. The glucose tolerance test was carried out after a 12 h fast by intraperitoneal injection with D-glucose (1 mg/g) in sterile saline. Blood was obtained from experimental mice by tail snip every 30 min, and blood glucose levels within 150 min were measured with One Touch SureStep test strips. Glucose tolerance was determined by quantification of the area under curve (AUC) for the glucose tolerance test (GTT).

### 4.4. Echocardiography

Transthoracic echocardiograms were performed by using a Vevo770 High-Resolution In Vivo Micro-Imaging System equipped with a RMV 710B scanhead (VisualSonics Inc., Toronto, Canada) on the ND and HFD-treated SIRT3 LoxP and SIRT ECKO mice. Cardiac function of the left ventricle was analyzed by High-Frequency Ultrasound Imaging software (VisualSonics Inc., Toronto, Canada). Briefly, the studied mouse was anesthetized via inhalation of 1.5–2% isoflurane mixed with 100% medical oxygen administered with a vaporizer in an isolated chamber for induction. Anesthesia was maintained with 1–1.5% isoflurane with a heart rate of ~400–450 beats per min (bpm). Ejection fraction (EF%) and fraction shortening (FS%) were measured by short-axis imaging in M-mode. LV diastolic function was measured by transmitral inflow Doppler. Pulsed-wave (PW) Doppler images were obtained in apical 4-chamber (A4C) view to measure the isovolumic relaxation time (IVRT), isovolumic contraction time (IVCT), and ejection time (ET) [6,36]. The myocardial performance index (MPI) was calculated from the following formula: MPI = (IVRT + IVCT)/ET. In addition, tissue Doppler images (TDI) were obtained from the mitral annulus to measure tissue motion velocity in early and late diastole (E’ and A’, respectively). To measure the coronary flow reserve (CFR), the left proximal coronary artery (LCA) was visualized in a modified parasternal LV short-axis view. The velocity of coronary flow at 1% of isoflurane was recorded as baseline by using PW Doppler. The isoflurane concentration was then increased to 2.5% for two minutes to induce hyperemia. The CFR was calculated as the ratio of the hyperemic peak diastolic flow velocity to baseline peak diastolic flow velocity [6,36,37]. Arterial stiffness was measured with noninvasive Doppler ultrasound [38]. The time difference between the peak aortic flow at two distinct locations of known distance in the aorta was measured to estimate the pulse wave velocity (PWV). PWV was calculated using the following equation: PWV = (distance between probes)/(∆time t_1_ − ∆time t_2_). 

### 4.5. Measurement of Blood Pressure by Tail-Cuff Method

The tail-cuff method was used to measure the mean blood pressure (MAP) and systolic pressure (SBP) in conscious mice by using an MC1000 BP Analysis System (Hatteras Instruments, Cary, NC, USA) at 10 weeks of age and after 20 weeks of HFD. The mice were trained and acclimated to restraint for 20–30 min for 4 consecutive days at the same time of day before the actual measurements.

### 4.6. Statistical Analysis

Data are presented as mean ± SEM. All data were tested for normality and passed the test. Statistical significance was determined using Student’s *t*-test (two-tailed) between means of two groups, or two-way ANOVA with Tukey’s post-hoc test as indicated, followed by Tukey’s post-hoc test (GraphPad Prism 8). *p* < 0.05 is considered statistically significant.
